# CAREGIVER SUPPORT RATIO IN EUROPE

**DOI:** 10.1093/geroni/igz038.500

**Published:** 2019-11-08

**Authors:** Oscar Riberio, Laetitia Teixeira, Lia Araujo, Constança Paúl

**Affiliations:** 1 University of Aveiro - Department of Education and Psychology | Center for Health Technology and Services Research (CINTESIS.UA), Aveiro, Portugal; 2 University of Porto - Institute of Biomedical Sciences Abel Salazar | Center for Health Technology and Services Research (CINTESIS), Porto, Portugal; 3 Escola Superior de Educação - Instituto Politécnico de Viseu | Center for Health Technology and Research (CINTESIS), Viseu, Portugal; 4 CINTESIS – Center for Health Technology and Services Research, Oporto, Portugal

## Abstract

The caregiver support ratio (CSR) has been defined as the number of potential caregivers aged between 45 and 64 (the most common caregiving age range) for each person aged 80 and over (subgroup of older adults most at risk of needing long term services and support). In 2010, for the USA, this number was calculated to be 7 to 1, a ratio that was projected to shrink to 4 to 1 in 2030, and to 3 to 1 in 2015 according to the AARP Public Policy Institute. In this study we used data from CENSUS HUB to calculate the CSR in Europe considering a total of 27 countries. Main results revealed that a group of Mediterranean countries (Italy, Greece, Spain and Portugal), along with France, Belgium and Sweden have the lowest CSR (5 to 1); on the other hand, the countries with the highest CSR are Slovakia (9 to 1), and Ireland, Poland, Cyprus and Malta (8 to 1). In average, for the 27 countries, the estimated number of caregivers per frail older person today is 6 to 1. These findings reveal important differences between countries and may inform EU policy decisions regarding long-term care (LTC). Given that informal care forms a cornerstone of all LTC systems in Europe, and that this continent faces a rapidly increasing number of people in very advanced age with extended years of disability living at home, estimating the CSR for the next decades is of crucial importance.

